# Investigation of the Effects of Dioctyl Sulfosuccinate on the Photodegradation of Benzo[a]Pyrene in Aqueous Solutions under Various Wavelength Regimes

**DOI:** 10.3390/molecules28155797

**Published:** 2023-08-01

**Authors:** Anthony M. Santana, Sadia Arif, Kristina Evteyeva, Fernando Barbosa, Andres D. Campiglia

**Affiliations:** 1 Department of Chemistry, University of Central Florida, 4111 Libra Dr., Orlando, FL 32816, USA; 2 ASTox Lab—Analytical and System Toxicology Laboratory, Department of Clinical Analyses, Toxicology and Food Sciences, School of Pharmaceutical Sciences of Ribeirão Preto, University of São Paulo, Avenida do Café s/n, Ribeirao Preto 14040-903, Brazil

**Keywords:** PAHs, benzo[a]pyrene, photodegradation, DOSS, surfactants

## Abstract

Due to the relatively high concentrations of polycyclic aromatic hydrocarbons (PAHs) in oil samples, oil spills in aquatic ecosystems release significant amounts of PAHs. Although remediation efforts often take place during or immediately after an oil spill incident, a portion of the released PAHs remains in the body of water. A natural phenomenon resulting from the direct exposure of PAHs to sunlight is photodegradation. This article investigates the effect of dioctyl sulfosuccinate (DOSS) on the photodegradation of benzo[a]pyrene (BaP), the most toxic PAH in the priority pollutants list of the US Environmental Protection Agency (EPA). DOSS is a surfactant typically used in the remediation of oil spills. Three lamps with maximum emission wavelengths at 350 nm, 419 nm, and 575 nm were individually and simultaneously used to irradiate aqueous solutions of BaP in the absence and the presence of DOSS. When irradiated with the 419 nm lamp or the 575 lamp, BaP showed no photodegradation. Upon irradiation with the 350 nm lamp and with the simultaneous use of the three lamps, the photodegradation of BaP followed first-order kinetics. Independent of the irradiation wavelength, the presence of DOSS increased the half-life of BaP in the aqueous solution. In the case of the 350 nm lamp, the rate constant of photodegradation in the absence and the presence of DOSS varied from (3.79 ± 0.97) × 10^−3^ min^−1^ to (1.10 ± 0.13) × 10^−3^ min^−1^, respectively. Under simultaneous irradiation with the lamps, the rate constant of photodegradation varied from (1.12 ± 0.35) × 10^−3^ min (no DOSS) to (3.30 ± 0.87) × 10^−4^ (with DOSS). Since the largest rate constants of photodegradation were observed in the absence of DOSS, the longer half-lives of BaP in the presence of surfactant were attributed to the incorporation of PAH molecules into the DOSS micelles.

## 1. Introduction

Polycyclic aromatic hydrocarbons (PAHs) are a group of fused-ring hydrocarbons known for their ubiquitous presence in the environment [1,2,3]. The most popular PAHs are known as “EPA-PAHs”, which consist of a set of 16 compounds included in the Environmental Protection Agency (EPA) priority pollutants’ list and usually monitored in water, air, and soil samples [4,5,6]. Some of the EPA-PAHs are known to be both toxic and carcinogenic [7,8,9]. Their ubiquitous environmental presence arises from natural and anthropogenic sources. These include forest fires, volcanic eruptions, and the incomplete combustion of fossil fuels [10,11].

Due to their relatively high concentrations in oil samples [12], incidents such as the Deepwater Horizon and Exxon Valdez events have released significant amounts of PAHs in aquatic ecosystems [13,14]. During oil spill incidents, rapid response takes priority, as spills typically require the remediation of millions of liters of oil that often span millions of square kilometers of water. Remediation efforts involve immediately removing as much of the oil as possible from the affected site. Chemical dispersants such as surfactants are often used to break up any oil still remaining in the contaminated site. The surfactants that are typically used in remediation efforts consist of long chain hydrocarbons featuring a hydrophilic region called the “head” and a hydrophobic region called the “tail”. A well-known example is dioctyl sulfosuccinate or DOSS, which lowers the surface tension of the oil slick to break up large areas of oil, allowing its dispersion into the water column and lowering toxicity through dilution.

In addition to removal from the environment via remediation, oil spills are subjected to sunlight exposure, a natural phenomenon that promotes the photodegradation of organic compounds in aquatic environments [15,16,17,18]. Numerous studies have been focused on the environmental factors that affect the photodegradation rates of PAHs via direct exposure to sunlight. These include the presence of hydroxy radicals and singlet oxygen [19], water salinity [20] and the sorption of PAHs onto organic materials [21]. The same is not true for the presence of surfactants remaining in aquatic environments from the anthropogenic remediation of oil spills. To the extent of our literature search, previous work on the remediation of PAHs with surfactants has been limited to soil samples [22,23].

Herein, we study the effect of DOSS on the photodegradation of benzo[a]pyrene (BaP) in aqueous solutions. BaP is the most toxic PAH in the EPA priority pollutants list and often used as a measure of contamination risk to human health [24,25]. Since surfactants reduce oil–water interfacial tension more efficiently when micelles are present on the surface of a waterbody [26], we investigate the effect of DOSS at a surfactant concentration slightly higher (8.0 mM) than its critical micellar concentration (CMC = 3.15 mM). The fluorescence intensities and the spectral profiles of BaP were monitored in the absence and presence of DOSS upon irradiation in a photochemical reactor equipped with lamps emitting maximum intensities at 350 nm, 419 nm and 575 nm. Upon irradiation with the 350 nm lamp and with the simultaneous use of the three lamps, the photodegradation of BaP followed first-order kinetics. Since the largest rate constants of photodegradation were observed in the absence of DOSS, the longer half-lives of BaP observed with the surfactant were attributed to the incorporation of BaP molecules into the DOSS micelles. The fact that micelle protection reduces the photodegradation of BaP in aqueous media should be taken into consideration for the future use of dispersants in oil spills.

## 2. Methods and Materials

### 2.1. Reagents

All chemicals were acquired at the highest purity available and used as received. BaP was purchased from Sigma Aldrich (St. Louis, MO, USA). DOSS was purchased from Arcos Organics. HPLC-grade methanol was purchased from Sigma-Aldrich. All water used in this study was of ultrapure quality and generated by a Barnstead Nano-Pure Infinity system (Barnstead, Dubuque, IA, USA).

### 2.2. Solution Preparation

Stock standard solutions of BaP were prepared in 100% methanol. DOSS solutions were prepared in water. Working solutions of BaP were prepared by diluting their stock solutions with either water or DOSS to a final methanol/water or methanol/DOSS volume/volume % ratio equal to 95%.

### 2.3. UV-Vis Absorption Spectroscopy

UV-Vis absorption spectroscopy measurements were performed with a Varian Cary-50 spectrometer (Agilent Technologies, Inc., Santa Clara, CA, USA) equipped with a 75 W Xenon lamp. All spectra were recorded using a 2 nm bandpass and 1 cm pathlength quartz standard cuvettes.

### 2.4. Room Temperature Fluorescence Spectroscopy

Fluorescence measurements were made with a Fluoromax-P spectrofluorometer (Horiba-Yvon, Irvine, CA, USA). This instrument was equipped with a 100 W Xenon lamp with an emission spectrum ranging from 200 nm to 1100 nm. The excitation and emission monochromators had a reciprocal linear dispersion of 4.2 nm∙mm^−1^ with a wavelength accuracy of ±0.5 nm with a 0.3 nm resolution. Their diffraction gratings were blazed at 330 nm for excitation and 500 nm for emission. Fluorescence detection was performed with a photomultiplier tube from Hamamatsu (model R928 from Horiba-Yvon) which had a spectral response from 185 nm to 650 nm and it was operated in the photon-counting mode. Instrument control was conducted with custom software (FluorESSENCE^TM^, version 3.8, Horiba Scientific, Piscataway, NJ, USA). 

Signal intensities and spectra were collected with micro-quartz cuvettes having a 1 cm pathlength, 2 mm width, and a maximum volume of 400 uL. All measurements were made from un-degassed solutions. A long-pass filter with a cutoff wavelength at 370 nm was used to remove scattered excitation light as well as second-order emission.

### 2.5. Photodegradation Studies

Irradiation of liquid solutions was carried out in a commercial photochemical reactor (Rayonet RPR-1000, The Southern New England Ultraviolet Co., Branford, CT, USA). Three lamps with maximum emission wavelengths at 350 nm (RPR 3500), 419 nm (RPR 4190), and 575 nm (RPR 5750) were used for this study. All lamps were purchased from The Southern New England Ultraviolet Co. All irradiations were targeted at 7 mL of solutions placed into glass vials fitted with threaded caps. Solutions were divided into two groups: exposed and dark control. Dark control vials were wrapped in aluminum foil to prevent any irradiation light from entering the vials. Two vials per group were simultaneously exposed during each experimental run. Sampling of the vials was performed in 30 min intervals for the first 3 h of exposure, followed by 1 h samplings for the next 4–8 h of exposure. Each sampling consisted of mixing 500 μL from each vial into an amber vial, followed by pouring the resulting solution into a micro-cuvette for fluorescence measurements with the spectrofluorometer.

## 3. Results and Discussion

### 3.1. Absorption and Fluorescence Characteristics of BaP

**Figure 1:**
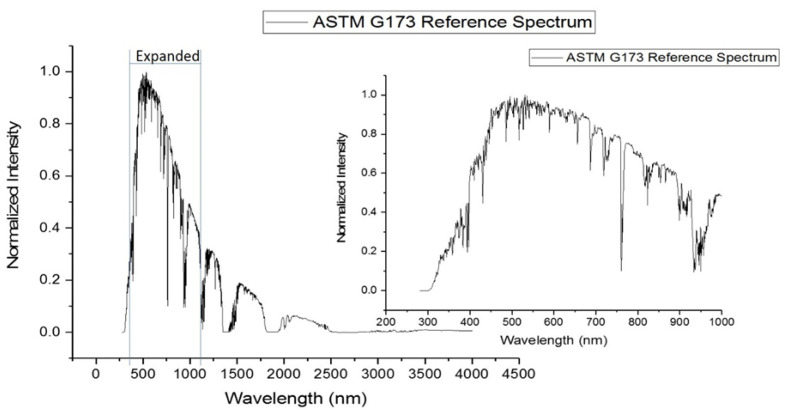
ASTM standard for solar radiance at sea level [27]. Expanded region shows irradiance profile within UV-Vis region of spectrum.

**Figure 2:**
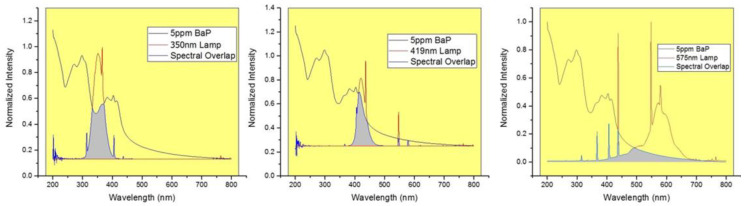
Spectral overlap of lamp emission profiles used for the irradiation experiments with the absorption spectrum of BaP.

Figure 1 shows the spectrum of terrestrial solar irradiance from the ASTM G173 standard, inlaid with an expanded scale for the region of highest intensity, [27] which occurs approximately between 300 and 900 nm. Figure 2 overlays the absorption spectrum of BaP with the emission spectra of the three irradiation lamps selected for this study. Table 1 summarizes the percentages of spectral overlapping for each irradiation lamp along with the absorptivity of BaP at the maximum emission wavelength of each lamp. The tabulated values show the largest overlap and the highest absorptivity for the irradiation lamp emitting the maximum intensity at 350 nm.

Figure S1 shows the excitation and fluorescence spectra of BaP recorded from a 99.5/0.5 *v*/*v* water/methanol solution. While the maximum emission wavelength of the 350 nm lamp is located at higher energy than the maximum excitation wavelength of BaP (365 nm), the maximum emission wavelengths of the other two lamps (419 nm and 575 nm) are located at lower energies than 365 nm. Table S1 summarizes the analytical figures of merit of BaP obtained from fluorescence measurements at their maximum excitation and emission wavelengths. All standard solutions were prepared in water/methanol at 99.5/0.5 *v*/*v*. A medium linear concentration (50 ng·mL^−1^) was then selected for all further studies with both compounds.

### 3.2. Irradiation Studies in the Absence of DOSS

The effect of irradiation time on the fluorescence characteristics of BaP was first investigated in the absence of DOSS. Individual PAH solutions were prepared in water/methanol (99.5/0.5; *v*/*v*) at a 50 ng·mL^−1^ concentration. Solutions were divided into two groups: exposed and dark control. Dark control vials were wrapped in aluminum foil to prevent any irradiation light from entering the vials. Both types of solutions were simultaneously exposed to the individual emission of each lamp and then to the combined emission of the three lamps over a total of 480 min.

As shown in Figure S2 and Table S2, the temperature of the photochemical reactor remained relatively constant over the course of our experiments. The temperature with the three lamps appears to be slightly higher than the temperature with each one of the lamps, but all the temperature averages are statistically equivalent. Since BaP solutions in the dark control vials did not experience any significant changes in their fluorescence intensities and/or spectral profiles, the modifications observed from BaP in the exposed vials resulted from the sole effect of sample irradiation.

**Figure 3:**
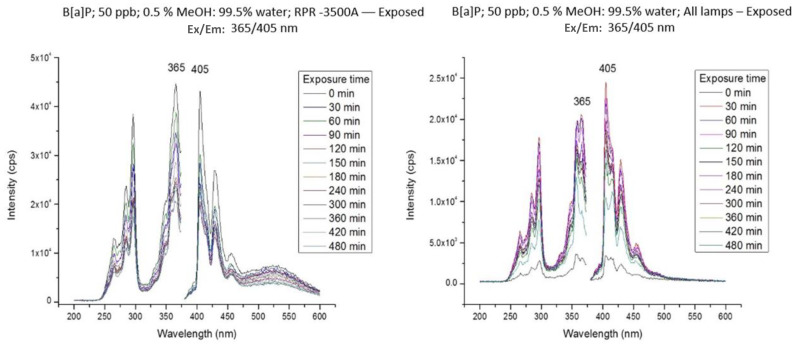
Excitation and fluorescence spectra of a 50 ng·mL^−1^ of BaP solution in methanol/water (0.5/99.5; *v*/*v*) as a function of irradiation time.

When irradiated with the 419 nm lamp or the 575 lamp, BaP showed no significant changes in its spectral features and fluorescence intensities. The effects of irradiation with the 350 nm lamp and with the simultaneous use of the three lamps are shown in Figure 3. In both cases, the fluorescence of BaP experienced significant intensity reductions as a function of irradiation time with no spectral modifications in its excitation and emission profiles. However, total PAH degradation did not occur during the total irradiation time (480 min). Since the spectral features remained constant over time and no additional fluorophores appeared during the entirety of the irradiation experiments, the photodegradation products of BaP appear to be non-fluorescent.

According to our literature search [28,29], the photodegradation of low concentrations of PAHs in aqueous solutions follows first-order kinetics according to Equation (1):

(1) $$ln[PAH]=ln[{PAH]}_{0}-kt$$

where [*PAH*]_0_ is the initial PAH concentration, [*PAH*] is the concentration at time *t*, and *k* is the first-order rate constant. For PAH concentrations within the linear dynamic range of the calibration curve, and fluorescence intensities measured at the maximum emission wavelength of the PAH, the terms [*PAH*] and [*PAH*]_0_ in Equation (1) can be substituted with the fluorescence intensities to obtain Equation (2):

(2) $$\ln\left[I_{t}\right]=\ln\left[I_{0}\right]-kt$$

where *I*_0_ and *I_t_* are the fluorescence intensities at time zero and time *t*, respectively. By rearranging Equation (2) into Equation (3),

(3) $$\ln\left[\frac{I_{t}}{I_{0}}\right]=-kt$$

while the rate constant of photodegradation (*k*) can be obtained from the slope of the plot ln[*I_t_*/*I*_0_] versus *t*, and the half-life of the irradiated *PAH* can be determined via Equation (4):

(4) $$t_{1/2}=\frac{\ln2}{k}$$

Figure S3 depicts the ln[*I_t_*/*I*_0_] versus t plots obtained for BaP under irradiation with the 350 nm lamp and with the combination of the three lamps. First-order kinetics plots were obtained in both cases. Table 2 summarizes the rate constants and the half-life values obtained for BaP in the absence of DOSS. Under irradiation with the three lamps, the photodegradation of BaP proceeded approximately three times slower than with the 350 nm lamp. As a consequence, the half-life of BaP upon irradiation with the 350 nm lamp was considerably shorter than its half-life upon irradiation with the three lamps. Since no photodegradation was observed under irradiation with the 419 nm and the 575 nm lamps, leading to the understanding that the longer half-life observed upon irradiation with the three lamps requires additional experiments beyond the scope of the present studies.

### 3.3. Irradiation Studies in the Presence of DOSS

The CMC of DOSS in methanol/water (0.5/99.5; *v*/*v*) was determined via the pyrene 1:3 ratio method [30]. The fluorescence spectra of pyrene were recorded at an excitation wavelength of 335 nm in the presence of various DOSS concentrations. Its fluorescence intensities were monitored at 371 nm (I_371_) and 384 nm (I_384_) to plot the I_371_/I_384_ ratio as a function of DOSS concentration. The obtained results are shown in Figure S4. The inflection point in the sigmoidal curve corresponds to the CMC of DOSS (3.15 mM). To ensure that all solutions had adequate micelle formation, all further studies were then performed with an 8 mM DOSS concentration.

**Figure 4:**
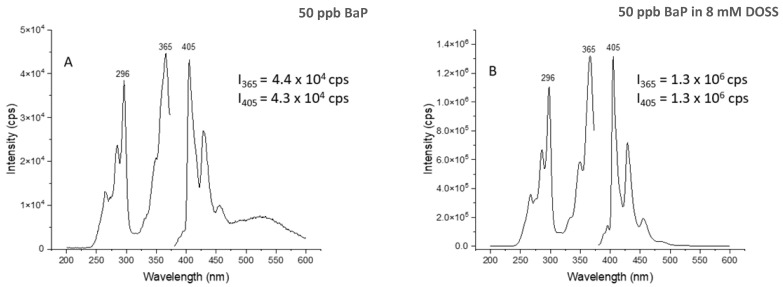
Spectral comparison of BaP in the absence (**A**) and the presence (**B**) of 8 mM DOSS. Intensities listed for the excitation and emission maximum represent the average of three determinations.

Figure 4 compares the fluorescence intensities of BaP in the absence and the presence of DOSS. Significant intensity increases were observed in the presence of DOSS. This observation confirms the formation of micelles at the 8 mM DOSS concentration. The fluorescence enhancements were due to the inclusion of BaP molecules into the DOSS micelles, which protected BaP molecules from collisional deactivation of the excited state [31].

Table 3 compares the rate constants and half-life values of BaP in the presence and the absence of DOSS. Similar to the experiments in the absence of DOSS, irradiation with the 419 nm or the 575 lamps caused no significant changes in the spectral features and the fluorescence intensities of this PAH. While its photodegradation was still observed in the presence of DOSS, the rate constant upon irradiation at 350 nm was approximately three times lower than the rate constant in the absence of DOSS. A similar decrease was observed upon the simultaneous irradiation of BaP with the three lamps. Considering the experimental data presented in Figure 4, it is reasonable to state that the incorporation of BaP into the DOSS micelles reduces, to some extent, its photodegradation in aqueous solutions.

## 4. Conclusions

Three lamps with maximum emission wavelengths at 350 nm, 419 nm, and 575 nm were individually and simultaneously used to irradiate aqueous solutions of BaP in the absence and the presence of DOSS. When irradiated with the 419 nm lamp or the 575 lamp, BaP showed no significant changes in its spectral features and fluorescence intensities. Upon irradiation with the 350 nm lamp and with the simultaneous use of the three lamps, the photodegradation of BaP followed first-order kinetics in the absence and the presence of DOSS. In the presence of DOSS, the rate constant upon irradiation at 350 nm was approximately three times lower than the rate constant in the absence of DOSS. A similar decrease was observed upon the simultaneous irradiation of BaP with the three lamps. Since the 8 mM concentration of the surfactant used in our studies was above the CMC of DOSS, the longer lifetime of BaP in the presence of surfactant was attributed to the incorporation of BaP molecules into the DOSS micelles. The fact that micelle protection increases the lifetime of BaP in aqueous media should be of note for the use of dispersants in environmental spills. Future photodegradation studies in our lab will investigate the effect of DOSS at concentrations below its CMC.

## Figures and Tables

**Table 1:** Absorption characteristics of BaP with the irradiation lamps used in these studies.

Irradiation Lamp ^a^ (nm)	350	419	575
**Percentage of Overlap ^b^ (%)**	16.4	10.7	11.9
**Absorptivity ^c^ (mL/µg∙cm)**	15,303 ± 1716	12,605 ± 3108	2921 ± 819

^a^ Maximum emission wavelength of irradiation lamp. ^b^ Percentage of overlap calculated with the formula [A_Overlap_/A_Total_] × 100, where A_Overlap_ and A_Total_ are obtained by integrating the overlapped and total areas under the PAH spectrum, respectively. ^c^ Calculation with the formula A/b∙c, where A is the absorptivity of BaP at the maximum emission wavelength of the lamp, b is the pathlength of the cuvette used for absorption measurements and c is the concentration of BaP. The averages are based on individual measurements of three aliquots.

**Table 2:** Rate constants (*k*) and half-lives (*t*_1/2_) obtained for BaP in the absence of DOSS ^a^.

BaP ^b^	Lamp Wavelength (nm)			All Lamps Together
	350	419	575	
***k*** (**min^−1^**)	(3.79 ± 0.97) × 10^−3^	ND ^c^	ND ^c^	(1.12 ± 0.35) × 10^−3^
***t*_1/2_ (min)**	182.9 ± 46.62	ND ^c^	ND ^c^	616.4 ± 189.8

^a^ All measurements made at the maximum excitation and emission wavelengths of BaP (365 nm/405 nm). ^b^ BaP solutions prepared in methanol/water (0.5/99.5; *v*/*v*) at a 50 ng·mL^−1^ concentration. ^c^ ND = no photodegradation was observed.

**Table 3:** Effect of DOSS on the rate constants (*k*) and half-lives (*t*_1/2_) of BaP.

BaP ^a^	Lamp Wavelength (nm)		All Lamps Together	
	350			
	No DOSS	DOSS	No DOSS	DOSS
***k*** (**min^−1^**)	(3.79 ± 0.97) × 10^−3^	(1.10 ± 0.13) × 10^−3^	(1.12 ± 0.35) × 10^−3^	(3.30 ± 0.87) × 10^−4^
***t*_1/2_** (**min**)	182.9 ± 46.62	633 ± 73	616.4 ± 189.8	2099.5 ± 554.2

^a^ All measurements made at the maximum excitation and emission wavelengths of BaP (365 nm/405 nm). BaP solutions prepared in 8 mM DOSS (methanol/water ay 0.5/99.5; *v*/*v*) at a 50 ng·mL^−1^ concentration.

## Data Availability

Data is contained within this article and supplementary material.

## Supplementary Materials

The following supporting information can be downloaded at https://www.mdpi.com/article/10.3390/molecules28155797/s1. Figure S1: Excitation and Fluorescence Spectra of BaP in 99.5/0.5 Water/Methanol. PAH concentration was 50 ng·mL^−1^.; Table S1: Analytical Figures of Merit for BaP; Figure S2: Sample temperature upon irradiation in photochemical reactor using the following: (A)350 nm lamp; (B) 419 nm lamp; (C) 575 nm lamp; and (D) the three lamps simultaneously (350 nm + 419 nm + 575 nm); Table S2: Average Temperature ^a^ of BaP Solutions Under Irradiation in the Photochemical Reactor.; Figure S3: First order kinetics graphs for the irradiation of 50 ng·mL^−1^ BaP in methanol/water (0.5/99.5; *v*/*v*) with the 350 nm lamp and with the three lamps; i.e., 350 nm, 419 nm and 575 nm.; Figure S4: Determination of the CMC of DOSS in methanol:water (0.5/99.5 *v*/*v*).
