# Supplementary material for: Investigation of the Effects of Dioctyl Sulfosuccinate on the Photodegradation of Benzo[a]Pyrene in Aqueous Solutions under Various Wavelength Regimes
Source: Molecules. 2023 Aug 1;28(15):5797. doi: 10.3390/molecules28155797 (PMC10420635; doi:10.3390/molecules28155797)
Supplement: Supplementary file 1 [file molecules-28-05797-s001.zip › molecules-2420520-supplementary.pdf]

Supplementary material

Investigation of the Effects of Dioctyl Sulfosuccinate on the Photodegradation of Benzo[a]Pyrene in Aqueous Solutions Under Various Wavelength Regimes

Anthony M. Santana [1], Sadia Arif [1], Kristina Evteyeva [1], Fernando Barbosa Jr. [2], and Andres D. Campiglia [1,*]

[1] Department of Chemistry, University of Central Florida, 4111 Libra Dr., Orlando, FL 32816-2366, USA; antmsantana@knights.ucf.edu (A.M.S.); sadia.arif@knights.ucf.edu (S.A.); kevteyeva@knights.ucf.edu (K.E.)
[2] ASTox Lab—Analytical and System Toxicology Laboratory, Department of Clinical Analyses, Toxicology and Food Sciences, School of Pharmaceutical Sciences of Ribeirão Preto, University of São Paulo, Avenida do Café s/n, Ribeirao Preto, SP 14040-903, Brazil; fbarbosa@fcfrp.usp.br
* Correspondence: andres.campiglia@ucf.edu; Tel.: +1-407-823-4162

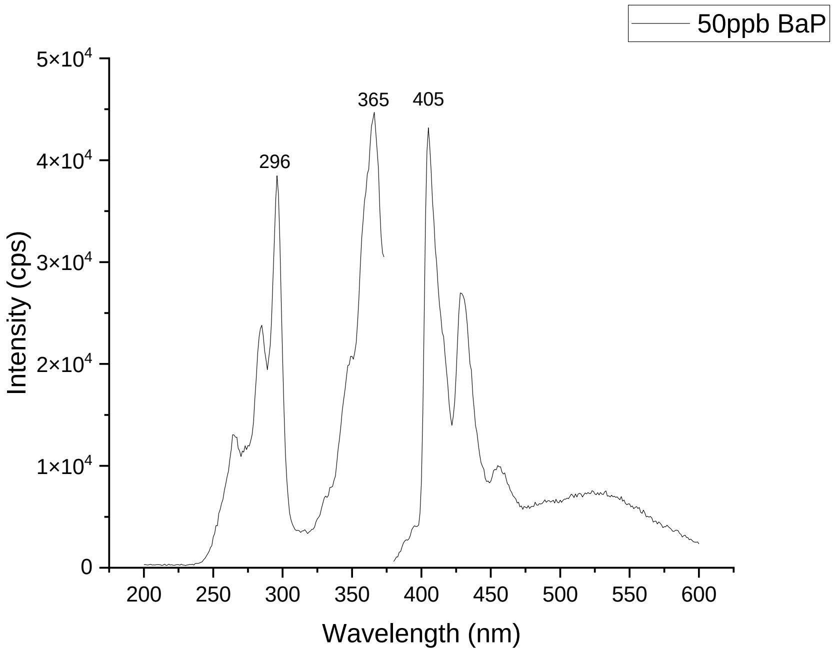

Figure S1. Excitation and Fluorescence Spectra of BaP in 99.5/0.5 Water/Methanol. PAH concentration was 50 ng·mL^{-1}.

Table S1. Analytical Figures of Merit for BaP.

Compound	$\lambda_{ex}/\lambda_{em}$ (nm)	LOD[a] (ppb)	LOQ[b] (ppb)	LDR[c] (ppb)	RSD (%)
BaP	365/405	1.0	3.3	3.3-144	5.7

[a] Limit of detection was determined by the formula (3*σ)/B, where σ is the standard deviation of the blank and B is the slope from the regression line. [b] Limit of quantitation was determined by the formula (10* σ)/B. [c] Linear dynamic range was determined as the LOQ to the highest measured concentration. The maximum linear concentration was not determined for these experiments.

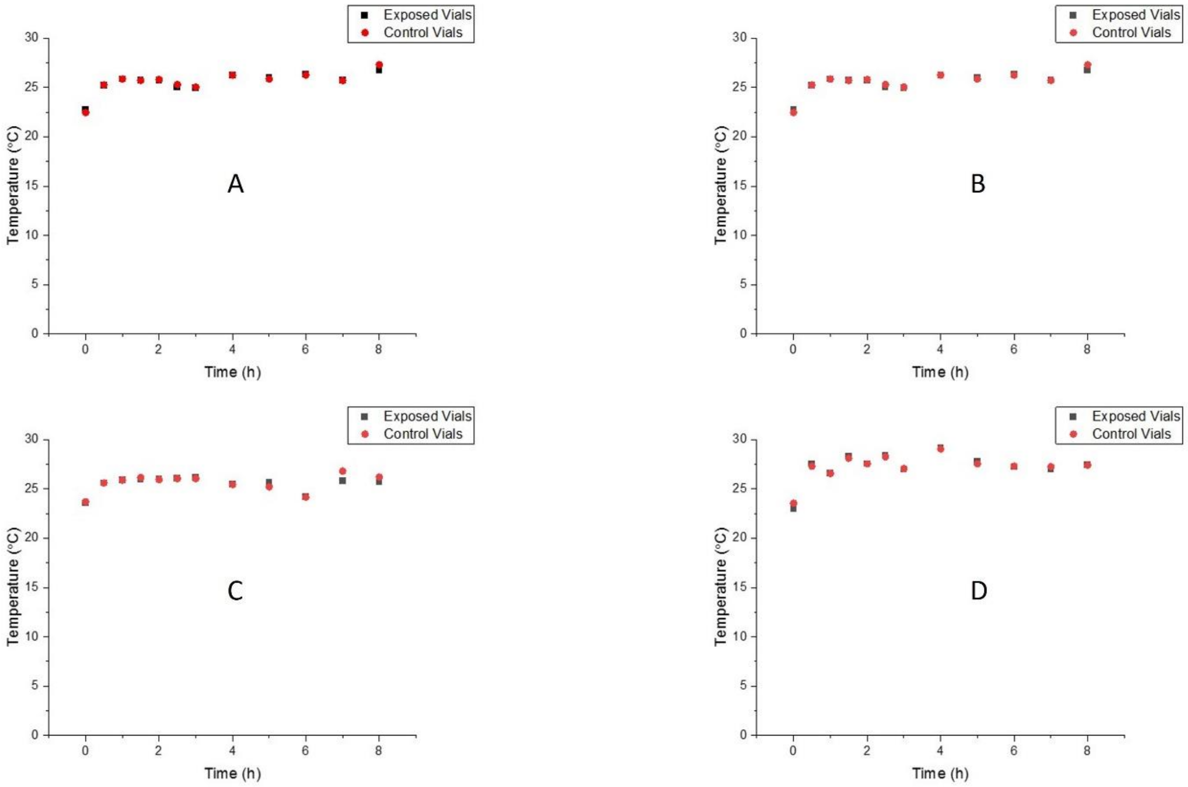

Figure S2. Sample temperature upon irradiation in photochemical reactor using the following: (A)350 nm lamp; (B) 419 nm lamp; (C) 575 nm lamp; and (D) the three lamps simultaneously (350 nm + 419 nm + 575 nm).

Table S2. Average Temperature [a] of BaP Solutions Under Irradiation in the Photochemical Reactor.

Lamp (nm)	Average Temperature Exposed Vials (°C)	Average Temperature Control Vials (°C)
350	25.5 ± 1.0	25.6 ± 1.1
419	25.7 ± 1.0	25.6 ± 0.9
575	25.5 ± 0.8	25.6 ± 0.8
All Lamps	27.2 ± 1.4	27.2 ± 1.3

[a] Averages are based on 12 measurements taken from each sample vial, with two vials for each vial type (exposed and control). Temperature was measured from each sample vial at 30-minute intervals from 0-3 hours of irradiation and 60-minute intervals for 4-8 hours of irradiation.

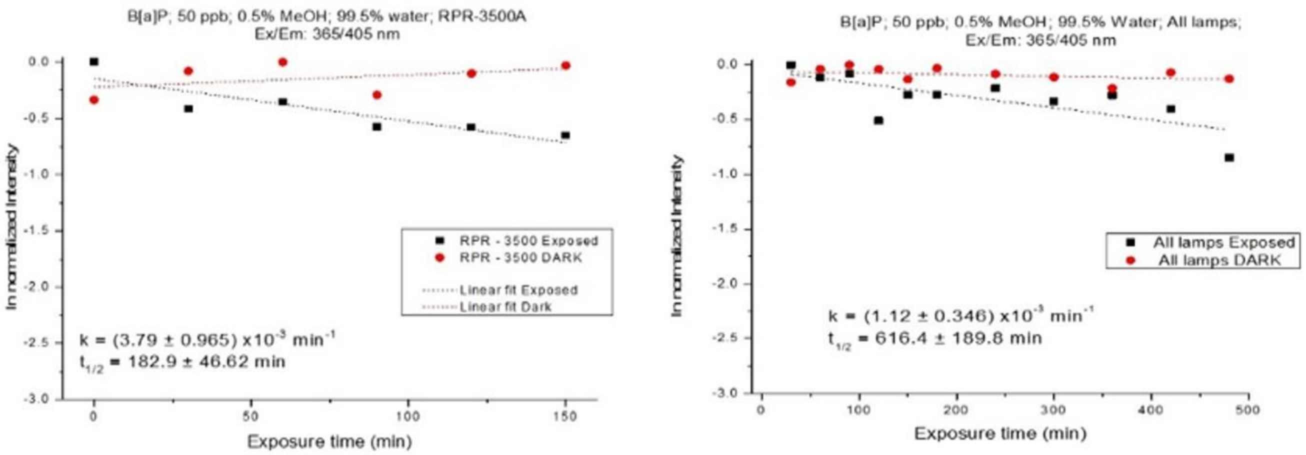

Figure S3. First order kinetics graphs for the irradiation of 50 ng.mL^{-1} BaP in methanol/water (0.5/99.5; v/v) with the 350 nm lamp and with the three lamps; i.e., 350 nm, 419 nm and 575 nm.

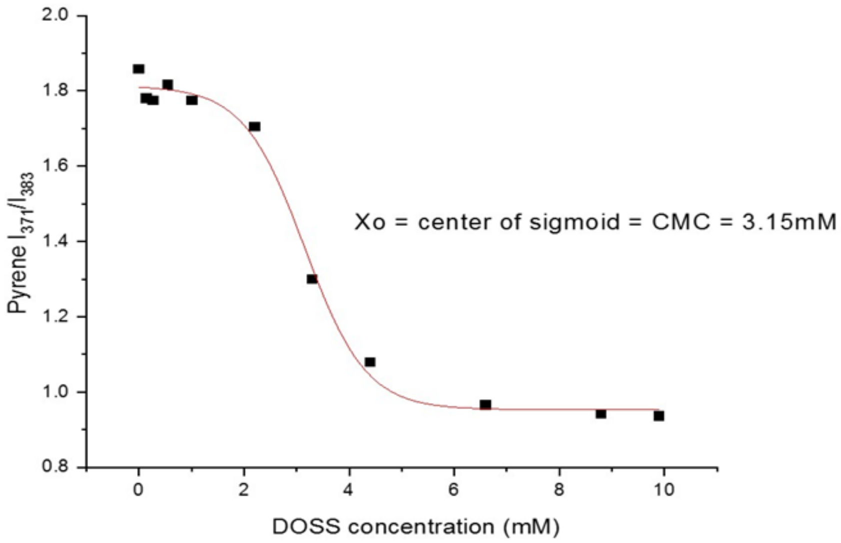

Figure S4. Determination of the CMC of DOSS in methanol:water (0.5/99.5 v/v).
